# Plasma proteomic characterization of the development of acute kidney injury in early sepsis patients

**DOI:** 10.1038/s41598-022-22457-w

**Published:** 2022-11-16

**Authors:** B. S. Star, C. K. Boahen, E. C. van der Slikke, V. M. Quinten, J. C. ter Maaten, R. H. Henning, V. Kumar, H. R. Bouma

**Affiliations:** 1 Department Clinical Pharmacy and Pharmacology, University Medical Center Groningen, University of Groningen, Groningen, The Netherlands; 2 Department of Internal Medicine and Radboud Institute of Molecular Life Sciences (RIMLS), Nijmegen, The Netherlands; 3 Department of Internal Medicine and Radboud Center for Infectious Diseases (RCI), Nijmegen, The Netherlands; 4 Department of Emergency Medicine, University Medical Center Groningen, University of Groningen, Groningen, The Netherlands; 5 Department of Internal Medicine, University Medical Center Groningen, University of Groningen, Groningen, The Netherlands; 6 Department of Genetics, University Medical Center Groningen, University of Groningen, Groningen, The Netherlands; 7 Nitte University Centre for Science Education and Research (NUCSER), Deralakatte, Mangalore, India

**Keywords:** Sepsis, Acute kidney injury

## Abstract

Acute kidney injury (AKI) develops frequently in the course of patients with sepsis and strongly associates with in-hospital mortality. However, diagnosing AKI involves a considerable lag-time because it depends on assessing an increase in serum creatinine, and offers no insight in the underlying pathophysiology. Consequently, identifying a set of proteins reflecting the development of AKI may improve earlier recognition of AKI and the understanding of its pathophysiology. A targeted plasma proteomic approach was performed in early sepsis patients with and without subsequent AKI development in a matched pair design (*n* = 19 each). Principal component analysis identified 53 proteins associated with development of AKI, which were further analysed using Enrichr gene ontology and pathway analysis. Nine differentially expressed proteins from the targeted proteomics were increased among patients who subsequently developed AKI and correlated with principal components, namely CALCA, CALR, CA12, CLEC1A, PTK7, KIM-1, NPPC, NUCB2 and PGF. We demonstrated the biological insight in the development of AKI in early sepsis compared to non-AKI sepsis.

## Introduction

Sepsis is a life-threatening syndrome of organ dysfunction encompassing a dysregulated host response to infection. The incidence of sepsis varies across the world and has been estimated at approximately 200 cases per 100,000 inhabitants in high-income countries, with an estimated in-hospital mortality rate of 17–26%^1–5^. The incidence of sepsis has more than doubled in the last decade and is expected to keep rising due to increased lifespan, chronic disease, use of immunosuppressive therapy, chemotherapy, and invasive procedures (e.g. mechanical ventilation, renal replacement therapy)^2,3^. Sepsis is frequently accompanied with organ dysfunction involving kidneys, liver, lungs, cardiovascular system and central nervous system. The incidence of AKI is approximately 22–51% among patients with sepsis and is strongly associated with subsequent failure of other organs and increased mortality. Moreover, patients with AKI have a higher risk for developing chronic kidney disease and end-stage renal disease, as well as long-term mortality after sepsis^4–12^.

The pathophysiology of sepsis-AKI is complex and incompletely understood, which both hampers prompt recognition of AKI and the development of novel treatments to prevent or treat AKI. The KDIGO criteria are commonly used to diagnose and classify AKI^13,14^. Importantly, the KDIGO criteria depend on measurement of urine output, which is usually lacking at and during early hospitalization, and changes in serum creatinine, which induces considerable lag-time in diagnostics because of the time needed to accumulate above the threshold^13^. Moreover, a recent pre-existent serum creatinine to determine the change in serum creatinine, is often not available. Here, we used targeted plasma proteomics to analyse a set of proteins involved in the pathophysiology of early sepsis, that contribute to the development of AKI and offer insight into the pathophysiology of AKI development. To this end, we performed a targeted proteomic analysis of plasma obtained from patients with early sepsis admitted to the emergency department (ED), either with or without subsequent development of AKI according to the KDIGO criteria.

## Methods

### Study population

Data were collected at the emergency department of the University Medical Center Groningen (UMCG), a tertiary care hospital with approximately 34,000 emergency department visits per year. Adult non-trauma patients (> 18 years of age) diagnosed with sepsis using the sepsis-2 criteria (i.e. two or more SIRS criteria combined with the clinical suspicion of an infection) with sepsis recognition and blood withdrawal within 30 min were included prospectively from 16th of August 2012 to 31st of June 2013 (*n* = 94). Development of AKI within the first 24 h was defined according to the KDIGO criteria^14^ by an increase of at least 50% of serum creatinine as compared to baseline creatinine or an absolute rise of more than 26.4 µmol/l (*n* = 19). Identified cases were matched with patients from the same cohort without AKI based on age, sex, infection focus and sepsis-2 criteria. Finally, technical exclusion of 2 samples during plasma proteomics resulted in a non-AKI group of 19 patients and an AKI group of 17 patients. Written (deferred) informed consent was obtained from all participating patients. The study protocols were approved by the medical ethical committee of the University of Groningen (METc 2012/077).

### Data collection

Collected data included patient characteristics, vital parameters and laboratory measurements at admission, and mortality at follow-up. Follow-up mortality was obtained from the Municipal Personal Records Database, containing full registration of all Dutch citizens. Patient characteristics consisted of age, gender, presence of diabetes mellitus, chronic kidney disease, liver disease, active cancer (radiotherapy or chemotherapy treatment received up to two years prior to the current hospitalization), and immunocompromised status. Vital parameters consisted of heart frequency, systolic and diastolic blood pressure, mean arterial pressure, body temperature (tympanic), oxygen saturation, and respiratory rate at admission. The recorded laboratory measurements consisted of haemoglobin, leukocyte count, thrombocyte count and plasma levels of lactate, creatinine, urea, and C-reactive protein (CRP). Other data included the source of infection, and length of hospital and intensive care unit (ICU) stay. Baseline serum creatinine and urea values (most recent measurement during the year preceding inclusion in the study) and those during hospitalization were collected from the electronic patient records. The estimated glomerular filtration rate (eGFR) was determined using the MDRD-4 formula^15^.

### Plasma proteome analysis

Plasma protein expression was assessed using the aptamer-based multiplex proximity extension assay (PEA) from Olink Proteomics AB (Uppsala, Sweden), allowing for quantification of a substantial number of proteins in a small amount of plasma^16^. In this assay, proteins are recognised by pairs of oligonucleotide-labelled antibodies (“probes”). When the two probes are in close proximity, a new PCR target sequence is formed by a proximity-dependent DNA polymerisation reaction, resulting in relative numbers. The resulting sequence is subsequently detected and quantified using a standard real-time PCR. Proteins from the Organ Damage panel were measured, containing 92 different proteins. Data from 25 protein analytes were omitted from the analysis, as the measured levels were below the LOD in ≥ 25% of the patients. Next, data were subjected to Olink proteomics performed quality control per sample during which samples that deviate less than 0.3 NPX from the median pass the quality control. Two individuals had to be excluded, as these failed quality control (QC) analysis by the manufacturer. Final data analysis comprised 67 protein analytes from 36 patients (*n* = 17 AKI and *n* = 19 without AKI).

### Enzyme-linked immuno-absorbent analysis (ELISA)

To perform a technical validation, we measured plasma levels of kidney injury molecule-1 (KIM-1), a biomarker for human renal proximal tubule injury, by ELISA (Quantikine; DSKM100). Results were read at an absorbance of 450 nm with wavelength correction at 570 nm using a Bio-Tek Synergy H4 microplate reader.

### Differential expression analysis

Differential expression analysis of identified proteins between AKI and non-AKI groups were performed using the R package limma, applying a linear model with age and sex as covariates. Limma uses an empirical Bayes method to moderate the standard errors of the estimated log fold changes^17^*.*

### Gene ontology and pathway enrichment analysis

Molecular function and pathway analysis were performed by Enrichr enrichment analysis. Enrichr for gene ontology (GO) molecular function and the NCI-Nature 2016 pathways analytic tools^18,19^. Enrichr is a comprehensive resource for composed gene sets and a search engine that accumulates biological knowledge for further biological discoveries. Proteins from PC1 and PC2 analyses with a fold change > 1 between sepsis and sepsis-AKI were used to identify GO and pathways (n = 48), and were used to perform GO and pathways analyses. P-values were calculated using Fisher exact test and corrected for multiple testing by Benjamini–Hochberg adjustment and pathways with a corrected *p*-value < 0.05 were considered significant. The combined score of the pathway analysis (Table 5) is calculated using the Fisher exact test and the Z-score to order the pathways.

### Statistical analysis

Statistical analyses were performed using SPSS 23 (IBM, USA). Normality was tested using a Shapiro–Wilk test. Since most data was non-normally distributed, categorical variables were compared using a using a z-test, while a Mann–Whitney *U* or Kruskal–Wallis test was used for continuous variables. PC were extracted from the principal component analysis (PCA) based on an Eigenvalue cut-off > 1, no rotation method was applied. Factors that correlated with the PCs, using a two-tailed Spearman test, were selected for further analysis. The correlation between proteomics and KIM-1 measurements were calculated using a two-tailed Pearson test. To identify factors associated with the course of renal function, PCs that were significantly different between patients with AKI and without AKI were entered in a stepwise linear regression analysis along with age, gender, sepsis severity as well as serum creatinine at baseline and upon admission (probability-of-F-to-enter < /= 0.05, probability-of-F-to-remove > /= 0.1). The Benjamini–Hochberg false discovery rate correction method was used where applicable to correct for multiple comparison testing. A *p*-value < 0.05 was considered significantly different.

### Statement of methods used

All methods were carried out in accordance with relevant guidelines and regulations.

### Statement of approval

The protocol was reviewed by the ethical review board of the UMCG and approved (METc 2012/077). Written informed consent was obtained from all participating patients.

## Results

### Characterisation of the early sepsis groups

From August 2012 to June 2013, we included in total 94 patients with sepsis at the ED. Of those patients, 19 (20%) developed AKI according to the KDIGO criteria in the first 24 h after admission. These 19 patients were matched with 19 sepsis patients without AKI from this cohort. Patients who later on developed AKI had a higher systolic blood pressure upon presentation to the ED, while mean arterial and diastolic blood pressure were not different from patients without subsequent development of AKI (Table 1). Other baseline and disease characteristics were not different between both groups. Upon admission, patients that subsequently developed AKI had increased serum creatinine and urea and decreased eGFR. Importantly, pre-existent renal function and the renal function at hospital discharge was not different between both groups (Table 2). Hence, at hospital admission, patients with subsequent sepsis-AKI only displayed decreased renal function and higher systolic blood pressure as compared to patients without AKI development.

**Table 1:** Baseline and disease characteristics.

	No AKI (*n* = 19) (median ± IQR) or n (%)	AKI (*n* = 17) (median ± IQR) or n (%)
Age (years)	65 (59–75)	60 (52–76)
Sex (male)	12 (63%)	13 (76%)
History of chronic kidney disease	6 (32%)	7 (41%)
History of cardiovascular disease	13 (68%)	10 (59%)
History of diabetes mellitus	9 (47%)	7 (41%)
Infection focus: airways/pneumonia	6 (32%)	5 (29%)
Infection focus: urogenital	9 (47%)	7 (41%)
Infection focus: abdominal	2 (11%)	3 (18%)
Infection focus: unknown	2 (11%)	2 (12%)
Systolic blood pressure (mmHg)	132 (125–150)	105 (90–130)*
Diastolic blood pressure (mmHg)	70 (65–80)	66 (52–80)
Mean arterial pressure (mmHg)	93 (88–107)	78 (67–97)
Heart frequency (bpm)	110 (95–120)	110 (100–125)
Respiratory frequency (per minute)	22 (18–28)	20 (18–29)
Oxygen saturation (%)	94 (91–98)	95 (90–97)
Glasgow coma score	15 (15–15)	15 (15–15)
Body temperature (°C)	38.5 (38.2–38.8)	38.6 (38.0–39.4)
Haemoglobin (mmol/L)	7.1 (6. 1–8.1)	7.2 (6.1–8.6)
Leukocytes (10^6^/mL)	12.2 (10–15.3)	8.5 (5–15.6)
Bandemia (> 5%)	2 (11%)	1 (6%)
Thrombocytes (10^9^/mL)	205 (174–317)	189 (146–217)
CRP (mg/L)	63 (22–197)	146 (80–335)
Lactate (mmol/L)	1.5 (1.1–1.7)	1.7 (1.2–2.1)
Multiple organ failure	3 (16%)	3 (18%)
Liver failure	0 (0%)	0 (0%)
Respiratory failure	6 (32%)	3 (18%)
ICU admittance	1 (5%)	3 (18%)
Length-of-stay in hospital (days)	6 (2–8)	8 (5–14)
Deceased in hospital	1 (5%)	1 (6%)
Oncology	2 (11%)	6 (35%)
Nefrotoxic drugs (i.e. cytostatics)	2 (11%)	5 (29%)

*IQR* interquartile range, *CRP* c-reactive protein, *ICU* intensive care unit, */**means p < 0.05/0.01.

**Table 2:** Course of renal function.

	No AKI (*n* = 19) (median ± IQR) or n (%)	AKI (*n* = 17) (median ± IQR) or n (%)
Baseline creatinine (μmol/L)	77 (62–102)	96 (84–132)
Baseline urea (mmol/L)	6 (5.3–8.4)	7.5 (6–10.0)
**Baseline eGFR (ml/min/1.73m**^**2**^**)**	90.7 (53.6–103.9)	68.4 (46.1–80.3)
CKD class: 1 (> 90)	10 (53%)	3 (18%)
CKD class: 2 (60–90)	4 (21%)	7 (41%)
CKD class: 3a (45–60)	1 (5%)	1 (6%)
CKD class: 3b (30–45)	0 (0%)	0 (0%)
CKD class: 4 (15–30)	3 (16%)	3 (18%)
CKD class: 5 (< 15)	1 (5%)	3 (18%)
Creatinine upon admission (μmol/L)	81 (65–105)	160 (104–222)**
eGFR upon admission (ml/min/1.73 m^2^)	80.9 (49.5–95.3)	40.3 (29.3–53.6)**
Urea/creatinine ratio at admission	90.2 (72.7–108.9)	76.4 (53.8–102.6)
Urea upon admission (mmol/L)	7.4 (5.5–10.3)	11.7 (8.9–16.5)*
Maximum creatinine < 48 h (μmol/L)	87 (66–105)	176 (118–222)**
Creatinine at discharge (μmol/L)	65 (57–90)	103 (77–134)
eGFR at discharge (ml/min/1.73m^2^)	88.3 (59.1–127.5)	69.6 (46.1–98.4)
Urea at discharge (mmol/L)	5.3 (3.7–8.6)	7.6 (6.8–9.4)
AKI at discharge	0 (0%)	1 (6%)
Creatinine after discharge (μmol/L)	65 (57–90)	103 (77–134)
eGFR after discharge (ml/min/1.73 m^2^)	88.3 (59.1–127.5)	69.6 (46.1–98.4)

Although no changes in baseline renal function existed between patients with and without AKI, the serum creatinine level, eGFR upon admission, as well as the maximum serum creatinine reached during the first 48 h after hospitalization are different between groups. *AKI* acute kidney injury, *CKD* chronic kidney disease, *eGFR* estimated glomerular filtration rate, *IQR* interquartile range, */**means p < 0.05/0.01.

### Targeted plasma proteomic revealed protein profiles involved in the development of AKI

**Figure 1:**
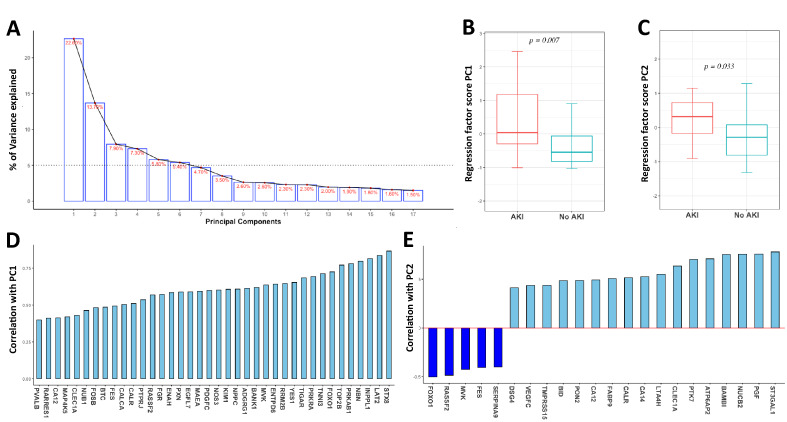
Summary of principal component analysis results (PCA). Result of the PCA, revealing approximately 23% of variance explained by PC1 (**A**). Analysis of all components that explained at least 5% variance (i.e. PC1–6) reveals a significant difference between patients with and without AKI for PC1 (**B**) and PC2 (**C**), but not for the other components. Extraction of the proteins associated with PC1 reveals 38 proteins (**D**), while 22 proteins were associated with PC2 (**E**). *AKI* Acute kidney injury, *PC* principal component, whiskers in box plot represent min–max.

**Figure 2:**
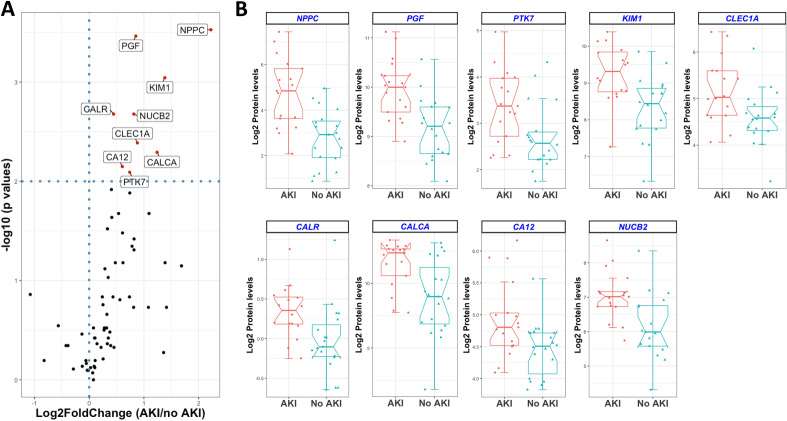
Plasma protein levels in sepsis patients with acute kidney injury as compared to patients without acute kidney injury. (**A**) Volcano plot demonstrates the log2 of the relative amount of plasma proteins and the − 10log p-value. Proteins with p-value < 0.01 are labelled in the volcano plot. (**B**) Boxplots demonstrating the nine upregulated proteins in patients with sepsis-AKI and sepsis. The boxplots represent the interquartile range of the protein levels and the whiskers represent the min/max range. *AKI* acute kidney injury.

In order to identify a set of proteins involved in the development of AKI in patients with sepsis, a targeted plasma proteomic analysis was performed. The organ damage panel measured 92 proteins; after deploying a missingness threshold of ≥ 25%, the panel identified 67 proteins*.* PCA of the proteins revealed 17 components, of which the first 6 had an Eigenvalue above the cut-off of 1. These 6 individual components explained from 25 to 5% of the variation of the development of AKI during sepsis (Fig. 1A). Of these 6 PCs, PC1 and PC2 demonstrated a positive regression coefficient between sepsis and sepsis-AKI (Fig. 1B,C). In total, 38 proteins correlated significantly with PC1 (Fig. 1D), while 22 proteins correlated with PC2 (Table 3; Fig. 1E). Of all identified proteins (*n* = 67), nine were increased among patients with sepsis-AKI as compared to sepsis (DEP fold change > 2, *p*-value < 0.05) and at the same time correlated with PC1, PC2 or both (Fig. 2A), namely: calcitonin gene related peptide 1 (CALCA), calreticulin (CALR), carbonic anhydrase 12 (CA12), c-type lectin domain family 1 member (CLEC1A), inactive tyrosine protein kinase 7 (PTK7), kidney injury molecule-1 (KIM-1), natriuretic peptide precursor C (NPPC), nucleobindin-2 (NUCB2) and prostaglandin F2 alpha receptor (PGF). Boxplots of these nine proteins in patients with sepsis-AKI and sepsis are depicted in (Fig. 2B). An additional validation of the proteomic analysis was confirmed by measuring KIM-1 (R = 0.517, p < 0.05). Thus, targeted proteomics revealed nine DEPs to play a role in the development of AKI during sepsis.

**Table 3:** Relative plasma levels of proteins extracted by principal component analysis.

Symbol	Uniprot ID	Full name	Correlation PCA	FC AKI vs. no AKI	p-value	Adj. p-value
ADGRG1	Q9Y653	Adhesion G-protein coupled receptor G1	PC1	1.48	0.156	0.285
ATP6AP2	O75787	Renin receptor	PC2	1.06	1.000	0.982
BAMBI	Q13145	BMP and activin membrane-bound inhibitor homolog	PC2	1.45	0.021	0.093
BANK1	Q8NDB2	B-cell scaffold protein with ankyrin repeats	PC1	1.26	0.433	0.547
BID	P55957	BH3-interacting domain death agonist	PC2	0.82	0.778	0.809
BTC	P35070	Probetacellulin	PC1	1.18	0.146	0.298
CA12	O43570	Carbonic anhydrase 12	PC1, PC2	**1.52**	0.007	**0.042**
CA14	Q9ULX7	Carbonic anhydrase 14	PC2	1.76	0.049	0.137
CALCA	P06881	Calcitonin gene-related peptide 1	PC1	**2.36**	0.005	**0.034**
CALR	P27797	Calreticulin	PC1, PC2	**1.36**	0.002	**0.022**
CLEC1A	Q8NC01	C-type lectin domain family 1 member A	PC1, PC2	**1.84**	0.004	**0.031**
DSG4	Q86SJ6	Desmoglein-4	PC2	1.37	0.471	0.567
EGFL7	Q9UHF1	Epidermal growth factor-like protein 7	PC1	1.26	0.030	0.106
ENAH	Q8N8S7	Protein enabled homolog	PC1	1.25	0.219	0.352
ENTPD6	O75354	Ectonucleoside triphosphate diphosphohydrolase 6	PC1	1.25	0.300	0.454
FABP9	Q0Z7S8	Fatty acid-binding protein 9	PC2	1.27	0.093	0.197
FES	P07332	Tyrosine-protein kinase Fes/Fps	PC1, PC2	1.07	0.754	0.816
FGR	P09769	Tyrosine-protein kinase Fgr	PC1	1.08	0.639	0.736
FOSB	P53539	Protein fosB	PC1	1.22	0.076	0.168
FOXO1	Q12778	Forkhead box protein O1	PC1, PC2	1.08	0.379	0.515
INPPL1	O15357	Phosphatidylinositol 3,4,5-trisphosphate 5-phosphatase 2	PC1	1.21	0.300	0.442
KIM1	Q96D42	Kidney Injury Molecule 1	PC1	**2.60**	< 0.001	**0.008**
LAT2	Q9GZY6	Linker for activation of T-cells family member 2	PC1	1.53	0.066	0.175
LTA4H	P09960	Leukotriene A-4 hydrolase	PC2	0.99	0.802	0.818
MAEA	Q7L5Y9	E3 ubiquitin-protein transferase MAEA	PC1	2.66	0.186	0.329
MAP4K5	Q9Y4K4	Mitogen-activated protein kinase kinase kinase kinase 5	PC1	0.68	0.285	0.444
MVK	Q03426	Mevalonate kinase	PC1, PC2	1.76	0.186	0.318
NBN	O60934	Nibrin	PC1	1.27	0.379	0.502
NOS3	P29474	Nitric oxide synthase, endothelial	PC1	1.77	0.038	0.119
NPPC	P23582	C-type natriuretic peptide	PC1	**4.67**	< 0.001	**0.010**
NUB1	Q9Y5A7	NEDD8 ultimate buster 1	PC1	1.16	0.471	0.555
NUCB2	P80303	Nucleobindin-2	PC2	**1.76**	0.002	**0.019**
PDGFC	Q9NRA1	Platelet-derived growth factor C	PC1	1.30	0.330	0.460
PGF	P43088	Prostaglandin F2-alpha receptor	PC2	**1.81**	< 0.001	**0.015**
PON2	Q15165	Serum paraoxonase/arylesterase 2	PC2	0.98	0.802	0.802
PRKAB1	Q9Y478	5′-AMP-activated protein kinase subunit beta-1	PC1	1.33	0.012	0.058
PRKRA	O75569	IFN-inducible dsRNA-dependent protein kinase activator A	PC1	1.02	0.754	0.799
PTK7	Q13308	Inactive tyrosine-protein kinase 7	PC2	**1.67**	0.008	**0.043**
PTPRJ	Q12913	Receptor-type tyrosine-protein phosphatase β	PC1	1.06	0.300	0.430
PVALB	P20472	Parvalbumin alpha	PC1	2.12	0.186	0.308
PXN	P49023	Paxillin	PC1	1.07	0.379	0.490
RARRES1	P49788	Retinoic acid receptor responder protein 1	PC1	1.33	0.023	0.087
RASSF2	P50749	Ras association domain-containing protein 2	PC1, PC2	0.96	0.684	0.771
RRM2B	Q7LG56	Ribonucleoside-diphosphate reductase subunit M2 B	PC1	2.13	0.021	0.086
SERPINA9	Q86WD7	Serpin A9	PC2	1.10	0.433	0.534
ST3GAL1	Q11201	CMP-N-acetylneuraminate-β-galactosamide-α-2,3-sialyltransferase 1	PC2	1.06	0.731	0.807
STX8	Q9UNK0	Syntaxin-8	PC1	1.53	0.033	0.110
TIGAR	Q9NQ88	Fructose-2,6-bisphosphatase	PC1	1.72	0.045	0.133
TMPRSS15	P98073	Enteropeptidase	PC2	1.67	0.146	0.287
TNNI3	P19429	Troponin I, cardiac muscle	PC1	3.22	0.071	0.164
TOP2B	Q02880	DNA topoisomerase 2-β	PC1	1.35	0.146	0.277
VEGFC	P49767	Vascular endothelial growth factor C	PC2	2.61	0.066	0.167
YES1	P07947	Tyrosine-protein kinase Yes	PC1	1.39	0.066	0.159

Principal component analysis revealed 38 proteins to be correlated with PC1 and 22 proteins to be associated with PC2, of which nine proteins are different between AKI as compared to non-AKI controls (bold). *AKI* acute kidney injury, *FC* fold change.

### Biological processes in the development of AKI during early sepsis

To analyse important pathways that were associated with the future development of AKI in sepsis, PCA proteins with a fold change > 1 were used to perform GO and pathway analyses using Enrichr. GO analysis showed an increase in the following molecular functions in patients with sepsis-AKI compared to matched pairs: immunoglobulin receptor binding (GO:0034987) (*p* = 0.041), carbonate dehydratase activity (GO:0004089) (*p* = 0.009) and platelet-derived growth factor receptor binding (GO:0005161) (*p* = 0.009) (Table 4). Further, Enrichr pathway analysis revealed nineteen pathways to be associated with sepsis-AKI (Table 5). Ranking the pathways using the combined scores demonstrated seven pathways above 200, revealing activation of endothelium (i.e. VEGF signalling, Angiopoietin receptor Tie2 and PDGF signalling). Together, Enrichr GO showed three molecular functions and nineteen biological pathways to be associated with sepsis-AKI, demonstrating an important role for endothelium in early prediction of progression towards sepsis-AKI.

**Table 4:** GO analysis molecular function by Enrichr.

Gene ontology	p-value	Adjusted p-value	Odds Ratio	Combined score
Immunoglobulin receptor binding (GO:0034987)	< 0.001	0.004	216.83	2034.63
Carbonate dehydratase activity (GO:0004089)	< 0.001	0.008	96.34	779.62
Platelet-derived growth factor receptor binding (GO:0005161)	< 0.001	0.009	78.82	610.51

Proteins that correlated with PC1 or PC2 and were increased among patients with AKI as compared to patients without AKI were used to identify pathways affected by sepsis-AKI.

**Table 5:** Pathway analysis by Enrichr/NCI Nature 2016.

Pathway name	p-value	Adjusted p-value	Odds ratio	Combined score
Signalling events mediated by VEGFR1 and VEGFR2	< 0.001	< 0.001	36.71	526.02
Angiopoietin receptor Tie2-mediated signalling	< 0.001	< 0.001	41.13	500.00
PDGF receptor signalling network	0.014	0.049	84.88	360.44
Class I PI3K signalling events	< 0.001	0.003	29.49	250.03
Alpha9 beta1 integrin signalling events	0.002	0.015	37.67	241.75
Glypican 1 network	0.002	0.015	34.66	217.05
Thromboxane A2 receptor signalling	< 0.001	0.004	25.03	200.74
Ephrin B reverse signalling	0.002	0.015	30.94	187.27
Regulation of p38-alpha and p38-beta	0.002	0.015	30.94	187.27
CXCR4-mediated signalling events	< 0.001	0.002	18.80	173.82
Alpha-synuclein signalling	0.003	0.016	28.87	171.07
EPHA forward signalling	0.003	0.016	27.07	157.11
Signalling events mediated by Hepatocyte Growth Factor Receptor (c-Met)	< 0.001	0.009	17.91	126.87
amb2 Integrin signalling	0.004	0.019	22.78	124.96
Signalling events mediated by PTP1B	0.007	0.030	17.31	86.05
Signalling events mediated by focal adhesion kinase	0.009	0.033	15.45	73.54
Fc-epsilon receptor I signalling in mast cells	0.009	0.033	15.45	73.54
PDGFR-beta signalling pathway	0.003	0.018	10.57	59.53
CDC42 signalling events	0.012	0.044	12.71	55.94

Proteins that correlated with PC1 and/or PC2 and which were positive differentially expressed proteins among patients with sepsis-AKI as compared to patients without AKI. The proteins were used to identify pathways affected by sepsis-AKI, only pathways with an adjusted p-value < 0.05 are shown.

## Discussion

Here, we used a targeted proteomic analysis of plasma derived from patients with sepsis admitted to the ED to explore the role of a set of proteins in the pathophysiology of sepsis-AKI. PCA revealed 53 proteins to be correlated with the development of sepsis-AKI, of which 48 proteins increased in sepsis-AKI and only 5 proteins decreased. Of these correlated proteins, nine also contributed to discrimination in PCA and were all significantly increased in sepsis-AKI (CLEC1A, CALR, KIM1, CA12, CALCA, NUCB2, PGF, PTK7 and NPPC). From the 48 proteins increased in sepsis-AKI, GO analysis demonstrated activation of immunoglobulin receptor binding, carbonate dehydratase activity and platelet-derived growth factor receptor binding in patients with sepsis-AKI. Enrichr pathway analyses assigned vascular endothelium activation and extracellular matrix remodelling during sepsis-AKI. The proteomic analysis of early sepsis patients at the ED demonstrated insights into pathways involved in the development of early AKI.

In the development of sepsis-AKI, PCA based GO analysis demonstrated three molecular functions of the proteins, associated with sepsis-AKI, namely immunoglobulin receptor binding, carbonate dehydratase activity and platelet-derived growth factor receptor binding. First, based on the molecular function (protein activity), the increased immunoglobulin receptors indicate altered immune cell activity by increased capacity of immune cells for immune regulatory functions. During sepsis-AKI, immune cells (i.e. neutrophils, macrophages and dendritic cells) infiltrate through the endothelial barrier to the kidney aggravating kidney injury by releasing ROS and proteases^20–22^. Second, given that carbonate dehydratase activity is mainly located inside the kidney and elevated during hypoxia, its increased circulating plasma levels likely indicate kidney injury because of increased circulating enzyme^23,24^. Third, the platelet-derived growth factor receptor binding exerts its stimulatory effects on cell growth, proliferation and fibrosis formation and it is released during kidney injury by endothelium and kidney cells^25,26^. Thus, GO indicates kidney injury by increased release of kidney carbonate dehydratase activity and increased vascular endothelial and kidney remodelling pathways via platelet-derived growth factor receptor binding during sepsis-AKI.

Pathway analyses support the role for endothelium in the development of AKI during sepsis^27,28^. The first three involved signalling events, namely VEGFR1 and VEGFR2, angiopoietin receptor Tie2-mediated signalling and PDGF receptor signalling network are mainly involved in vascular endothelial regulation. The pathophysiology of endothelium and kidney during sepsis-AKI is provoked by immune cells recruitment, leading to elevated ROS and cytokines release, abating endothelial function and aggravating sepsis-AKI^21,22,27,29^. Together, these pathways hint at a crucial role of endothelial signalling systems in subsequent development kidney dysfunction^27,28,30^. Further, the subsequent four signalling pathways, class I PI3K signalling events, alpha9 beta1 integrin signalling events, glypican 1 network and thromboxane A2 receptor signalling, reflect the response of endothelia and kidney cells due to tissue injury. Class I PI3K signalling is upregulated and has ambivalent effects in mediating kidney injury by supporting protective pathways in preclinical AKI models^31,32,33^, where Alpha9 beta1 integrin signalling events is used by endothelial and kidney cells to migrate and adhere to the extracellular matrix, thereby mediating remodelling and fibrosis formation, indicating the loss of functional endothelial and kidney tissue^34,35^. Circulating glypican 1 network originates from a dysfunctional glycocalyx, an endothelial barrier, associated with organ failure during infections. Sepsis-induced damage of the endothelial glycocalyx breaks down vascular barrier and contributes to microcirculatory changes in sepsis-AKI^36,37^. Further, an activated thromboxane A2 pathway causes platelet adhesion, capillary thrombosis and mediates vasoconstriction, decreasing kidney blood flow and function^38–40^. These findings are in line with other studies reporting the role of endothelial dysfunction in the development of sepsis-AKI^41,42,43^. Together, plasma proteomic profile of sepsis patients strongly suggest that endothelium dysfunction precedes development of acute kidney injury, suggesting that an endothelium protective strategy early in sepsis may protect from the development of sepsis-AKI.

We revealed underlying pathways involved in the development of AKI during sepsis compared to non-AKI sepsis. Current literature on sepsis-AKI described the microvascular network via leakage and signalling as critical contributor to the progression of kidney injury. The role of endothelial loss altering kidney function is thought to be due to hypoxia and endothelial barrier loss promoting extravasation of bacterial toxins and signalling molecules, in turn affecting underlying organs^27,44,45,46^. In this paper we emphasize the importance of the relation between endothelium and kidney function.

A potential limitation of the current study is that the organ damage panel is limited to quantification of 92 proteins. Further, the study design consists of a small cohort of 38 patients in a single tertiary hospital in the Netherlands, which allowed us to identify potential pathways involved in sepsis-AKI, but not to validate individual biomarkers. Proteins involved in the pathophysiology of AKI can after validation be selected for routine lab analyses, such as CRP. Additionally, we performed a pathway analysis on a targeted proteomic approach which may bias towards specific pathways (over)represented in the targeted proteomics. For future perspectives a larger cohort can be used to differentiate between transient and persistent AKI^47^. The values determined using the oLink multiarray are relative and, therefore, cannot be directly compared to the absolute values of different biomarkers or values obtained from other panels. Further, although our hospital serves a diverse patient population from a substantial geographical area of both urban and rural nature, this study was performed in an academic tertiary care teaching hospital, which may limit generalizability to other settings.

## Conclusion

Principal component analyses of the targeted proteomics revealed 53 proteins to be associated with the development of AKI among patients with early sepsis. Gene ontology (GO) of the proteins from PC1 and PC2 analyses with a fold change > 1, indicated increased immune antibody binding capacity, release of kidney carbonate dehydratase activity and platelet-derived growth factor receptor binding. Pathway analyses reported endothelial activation during kidney dysfunction in the pathophysiology of sepsis-AKI. Further, unravelling the pathophysiology of sepsis-AKI could be of major relevance to identify targets that enable early recognition or may serve to develop novel strategies to prevent the development AKI and optimize the outcome after sepsis.

## Data Availability

The datasets generated during and/or analysed during the current study are available from the corresponding author on reasonable request.
